# Challenging the Myth of Non-Response to the Ergogenic Effects of Caffeine Ingestion on Exercise Performance

**DOI:** 10.3390/nu11040732

**Published:** 2019-03-29

**Authors:** Juan Del Coso, Beatriz Lara, Carlos Ruiz-Moreno, Juan José Salinero

**Affiliations:** Exercise Physiology Laboratory, Camilo José Cela University, 28692 Madrid, Spain; blara@ucjc.edu (B.L.); cruizm@ucjc.edu (C.R.-M.); jjsalinero@ucjc.edu (J.J.S.)

**Keywords:** individual responses, responders, exercise performance, ergogenic aids

## Abstract

The ergogenicity of caffeine on several exercise and sport situations is well-established. However, the extent of the ergogenic response to acute caffeine ingestion might greatly vary among individuals despite using the same dosage and timing. The existence of one or several individuals that obtained minimal ergogenic effects or even slightly ergolytic effects after caffeine intake (i.e., non-responders) has been reported in several previous investigations. Nevertheless, the concept non-responding to caffeine, in terms of physical performance, relies on investigations based on the measurement of one performance variable obtained once. Recently it has been suggested that correct identification of the individual ergogenic effect induced by caffeine intake requires the repeated measurement of physical performance in identical caffeine–placebo comparisons. In this communication, we present data from an investigation where the ergogenic effect of acute caffeine intake (3 mg/kg) was measured eight times over a placebo in the same individuals and under the same conditions by an incremental cycling test to volitional fatigue and an adapted version of the Wingate cycling test. The ergogenic response to caffeine varied from 9% to 1% among individuals, but all participants increased both cycling power in the incremental test and Wingate mean power at least three to eight times out of eight the caffeine–placebo comparisons. These data expand the suggestion of a minimal occurrence of caffeine non-responders because it shows that all individuals responded to caffeine when caffeine is compared to a placebo on multiple and repeated testing sessions.

## 1. Introduction

2018 has been a prolific year for the publication of manuscripts aimed at explaining the causes of the interindividual variations for the ergogenic response of caffeine ingestion on exercise performance. Particularly, we read with interest the reviews by Southward et al. [1] and Fulton et al. [2] and the letter by Grgic [3], published in *Nutrients* in 2018, because they offered new insights towards unveiling the causes of the variability on physiological responses to caffeine. With this communication, we want to expand the understanding about why some individuals obtain less ergogenic benefits after the ingestion of a moderate dose of caffeine than others, and perhaps it will help to dispel the myth/concept of non-responders to caffeine, at least when referring to exercise performance. 

## 2. Individual Responses to Ergogenic Effects of Caffeine Ingestion

The utility of caffeine to increase physical performance in several exercise and sport situations is well-established and has been recently confirmed by systematic reviews and meta-analyses [4,5,6,7]. In addition, the use of caffeine or caffeinated products before competition is high, especially in individual sports or athletes of sports with an aerobic-like nature [8]. However, a small number of investigations have shown that the extent of the ergogenic response(s) to acute caffeine ingestion might greatly vary among individuals ([9,10,11] and the analysis of several investigations in [3]). These latter investigations have used cross-over and randomized experimental designs where the intake of a moderate dose of caffeine (1–6 mg/kg) is compared to a placebo condition in a group of individuals. Interestingly, these investigations indicated that, despite caffeine having produced an increase in physical performance as a group mean, one or several individuals obtained minimal ergogenic effects or even slightly ergolytic effects after caffeine intake despite being under the same experimental protocol. These individuals are frequently categorized as non-responders to the ergogenic effects of caffeine [12] and the causes for the lack of a positive physical response to caffeine have been associated to genetic (CYP1A2 and ADORA2A polymorphisms) and environmental factors, such as tolerance developed by chronic caffeine use and inappropriate timing and dose of administration or training status [13,14]. 

## 3. The Concept of Non-Responding to Caffeine Based on One Caffeine–Placebo Comparison

Recently, Pickering and Kiely [13] and Grgic [3] have criticized the concept non-responding to caffeine, in terms of physical performance, because this notion mostly relies on investigations based on the measurement of one performance variable obtained once. This experimental methodology to assess individual responses to caffeine ingestion might produce erroneous inferences because an individual does not always respond to caffeine to the same extent in all forms of exercise testing [9,15]. In addition, the reliability of the exercise test also needs to be considered when extrapolating conclusions regarding possible non-responses to the performance-enhancing effects of acute caffeine intake [3]. In fact, investigations where the ergogenic response to caffeine was explored by using the results of more than one physical performance test have shown that one participant might be categorized as a responder and a non-responder to caffeine at the same time due to his/her different outcomes in the different performance tests [9,15]. Pickering and Kiely [13] and Grgic [3] concur in suggesting that correct identification of the individual ergogenic effect induced by caffeine intake requires the repeated measurement of physical performance in identical caffeine–placebo comparisons. As suggested by Grgic [3], one of the following options can be selected to assess the individual ergogenic effect induced by caffeine: (1) multiple exercise tests with the same dose of caffeine or, (2) multiple doses of caffeine with the same exercise test, or (3) using a more complex protocol that combines repeated assessments of physical performance on different days using the same exercise test and dose of caffeine. If this is the case, most of the previous investigations on the study of individual responses to ergogenic effects of caffeine might not be methodologically correct because the categorization has been mainly based on one caffeine–placebo comparison. 

## 4. Repeated Testing of the Ergogenic Effect of Caffeine Ingestion Measured on Two Exercise Tests

We have recently published an investigation where the ergogenic effect of caffeine (3 mg/kg) was measured eight times over a placebo in the same individuals by using two physical performance tests: an incremental cycling test to volitional fatigue (25 W/minutes) and an adapted version of the Wingate cycling test [16]. The performance measurements were accompanied by the measurement of resting blood pressure, in addition to other physiological variables. The investigation was aimed at determining the time course of tolerance to the performance benefits of caffeine, and 11 participants ingested 3 mg/kg/day of caffeine, or a placebo, for 20 consecutive days. It is important to indicate that all participants were light caffeine consumers and refrained from all sources of dietary caffeine for the month before the onset of the experiment to eliminate the effect of habituation to caffeine (which represents another possible source of error when assessing individual responses). The caffeine–placebo comparisons were made after 1, 4, 6, 8, 13, 15, 18, and 20 days of consecutive caffeine or placebo ingestion while the order of the 20-day treatments was randomized. The coefficient of variation of the exercise tests and of the arterial blood pressure measurement were calculated by using the values obtained in the 20-day placebo treatment. A complete description of methods and standardizations can be found in the publication of this experiment [16]. 

**Figure 1:**
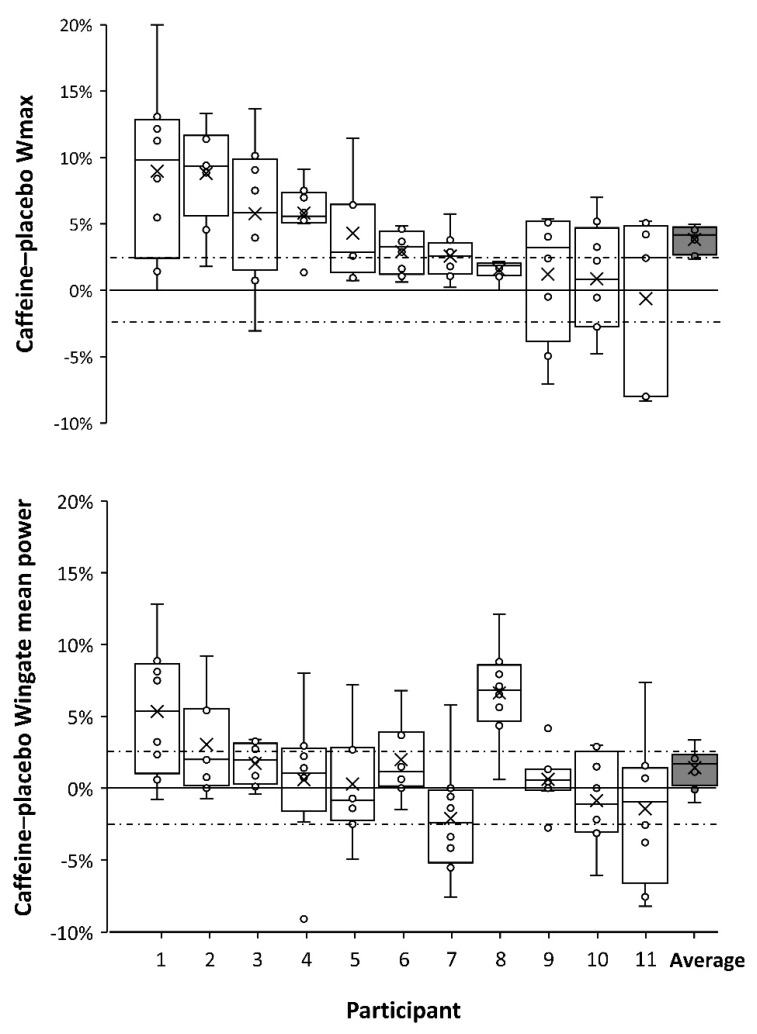
Box-and-whisker plots for the ergogenic effects of 3 mg/kg of caffeine on cycling power during a graded exercise test (upper panel) and during a 15-second Wingate test (lower panel). Caffeine was compared to a placebo on eight different occasions and each plot represents the results of these eight caffeine–placebo comparisons for each participant. “Average” represents the mean values for all 11 participants. The cross depicts the mean value for each individual while the lower, middle, and upper lines of the box represent the 25%, 50%, and 75% percentile for each individual. Whiskers represent the lowest and highest values (range). The black dashed line represents the natural variation of the graded exercise test (± 2.4%) and the 15-second Wingate test (± 2.7%) measured during the placebo treatment.

Because the tolerance to the ergogenic effect of caffeine was not completed after 20 days of consecutive ingestion, we have performed a sub-analysis for this communication to present the individual responses to acute caffeine intake in each of the eight identical caffeine–placebo comparisons. Figure 1 presents individual box-and-whisker plots for changes induced by caffeine intake, over the ingestion of a placebo, on cycling power obtained during the incremental test (Wmax) and mean cycling power obtained during the 15-second Wingate test. Figure 1 is a clear example of the interindividual variability in response to caffeine ingestion, with diverse caffeine-induced ergogenicity observed among individuals. Figure 1 has been organized in a ergogenicity-decrescent manner from left to right, with the participant showing the highest response to the ergogenic effects of caffeine at the left (subject 1 = 9.0 ± 3.6% and 2.3 ± 1.4% for Wmax and Wingate cycling power, respectively) and the individual with the lowest response at the right (subject 11 = 0.6 ± 6.3% and 1.6 ± 4.2% for Wmax and Wingate cycling power, respectively). Furthermore, Figure 1 also shows the intraindividual variability for the ergogenic effects of caffeine on both exercise performance tests. This figure disputes the notion of non-responding to the ergogenic effect of caffeine because all of the 11 included participants improved performance following caffeine ingestion, in either the graded exercise test or the Wingate test, in at least three testing occasions (with the magnitude of improvements exceeding the coefficient of variation for each test). These data expand the suggestion of a minimal occurrence of non-responders [3] because it shows that all individuals responded to caffeine, to an extent above the random error of the performance tests, when a repeated caffeine–placebo testing protocol was used to assess individual responses to caffeine. Thus, in the opinion of the authors of this manuscript, the concept of non-responders to the ergogenic effects of caffeine should be revisited. 

**Figure 2:**
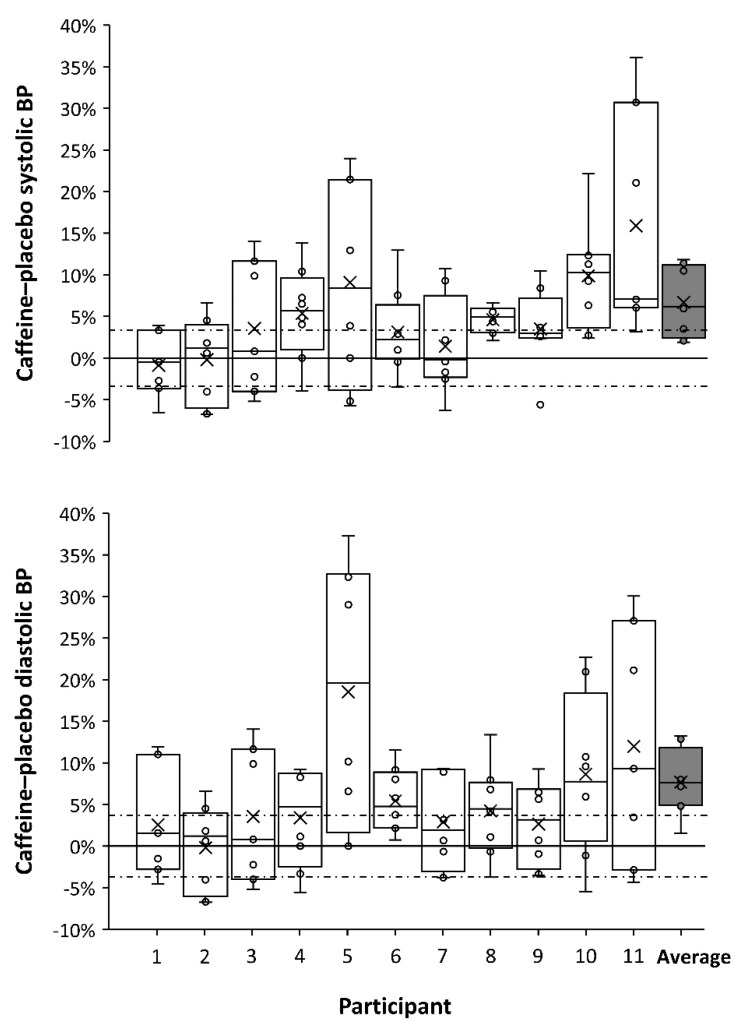
Box-and-whisker plots for the effects of 3 mg/kg of caffeine on resting systolic (upper panel) and diastolic (lower panel) blood pressure (BP). Caffeine was compared to a placebo on eight different occasions and each plot represents the results of these eight caffeine–placebo comparisons for each participant. “Average” represents the mean values for all 11 participants. The cross depicts the mean value for each individual while the lower, middle, and upper lines of the box represent the 25%, 50%, and 75% percentile for each individual. Whiskers represent the lowest and highest values (range). The black dashed line represents the natural variation of the systolic (± 3.3%) and diastolic blood pressure (± 3.8%) measured during the placebo treatment.

**Figure 3:**
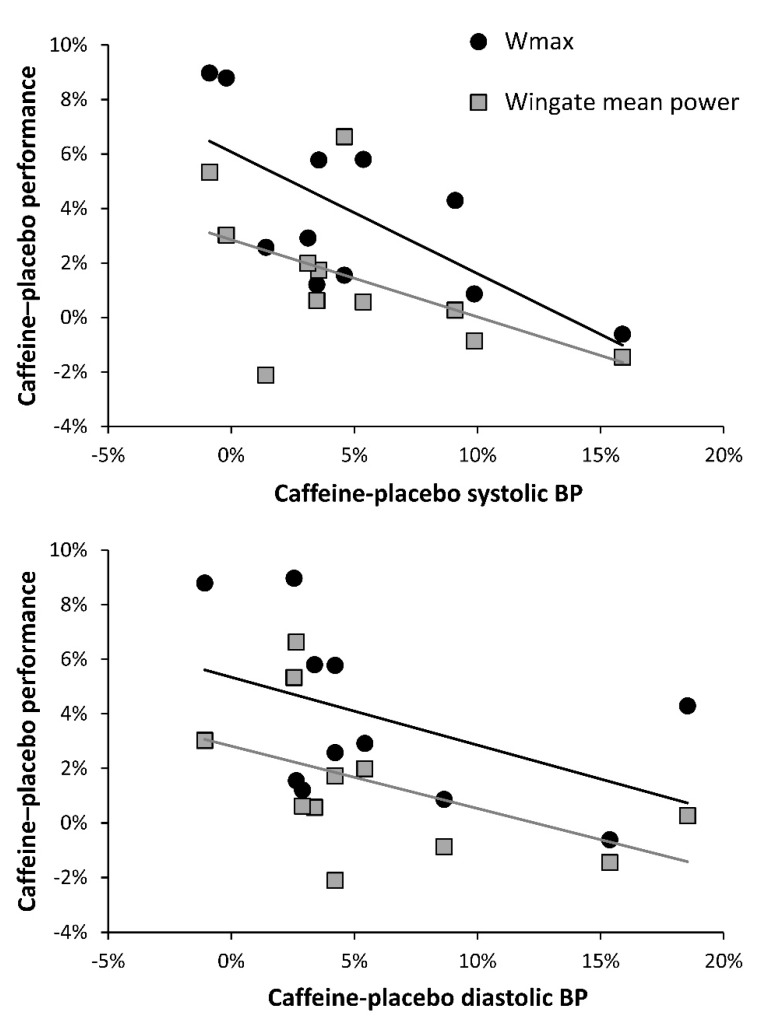
Relationships between the ergogenic effect of caffeine and systolic (upper panel) and diastolic (lower panel) blood pressure (BP). The ergogenic effect of caffeine was obtained by measuring peak cycling power during a graded exercise test (Wmax) and during a 15-second Wingate test. Caffeine was compared to a placebo on eight different occasions and each dot represents an average of these eight caffeine–placebo comparisons for each participant.

Figure 2 offers further insights on this topic because it presents individual data on caffeine-induced changes on resting systolic and diastolic blood pressure, measured before exercise, which is a variable also employed to categorize individual responses to acute caffeine ingestion [17]. As it happens with the ergogenic effect of caffeine, the outcomes of caffeine on blood pressure had great inter- and intraindividual variability. However, the participants with the highest responses to the cardiovascular effects of caffeine were the ones with the lowest response to the ergogenic effects of caffeine (with the exception of subject 5). To further explore this relationship, Figure 3 associates ergogenic and cardiovascular responses to caffeine ingestion. Interestingly, changes induced by caffeine intake in both systolic and diastolic blood pressures were negatively related to caffeine ergogenicity in both cycling performance tests. Briefly, this would mean that the individual with a high response to the cardiovascular effects of caffeine would be less prone to obtain ergogenic benefits from this substance. Although the mechanism behind this association is not evident from the current analysis, the association between high cardiovascular response to caffeine and decreased performance effects of caffeine has support in the literature. Wardle et al. [18] found that high cardiovascular responders to a 200-mg dose of caffeine decreased their willingness to exert an effort, a negative outcome that was not present in low cardiovascular responders to caffeine. This information might suggest that the cardiovascular and performance effects of caffeine might be incompatible and implies that high and low responders to the ergogenic effect of caffeine may exhibit divergent blood pressure response following acute caffeine ingestion. However, given the overall low sample number of the current study, this is an area that merits future research. If we can pinpoint that simple measurements such as blood pressure responses to caffeine ingestion are related to the magnitude of improvements in performance, this information may be of considerable practical importance for coaches and athletes when determining an optimal approach to caffeine supplementation. 

## 5. Conclusions

In conclusion, the data provided in this communication do not dispute the existence of a great interindividual variability to the ergogenic effects of caffeine ingestion, nor the genetic, environmental, or epigenetic causes associated to this variability. However, this analysis suggests that all individuals, to some extent, positively respond to the acute ingestion of 3 mg/kg of caffeine, while the magnitude of the ergogenic effect might be the result of the totality of consequences induced by caffeine ingestion on the human body. In this respect, this communication suggests that the individuals with a high response to the cardiovascular effects of caffeine would be less prone to obtaining ergogenic benefits from this stimulant. Caffeine ergogenicity might be subject to genetic influence, but future investigations on this topic should assess the individual ergogenic response to caffeine by using different forms of exercise testing and/or by using well-standardized caffeine–placebo comparisons on multiple, repeated testing sessions. In the point of view of the authors, this repeated measurement of the ergogenic effect of caffeine would help to reduce the equivocal findings of previous investigations on genetic variations [2]. From a practical perspective, the adjustment of appropriate dosage, timing, and form of administration of caffeine for an athlete might require several examinations in which physical performance and side-effects of caffeine should be measured and registered over a control situation. Gathering conclusions about the ergogenic effect of caffeine in one individual solely based on the results from one performance test might induce erroneous conclusions in both scientific and sport settings. The use of multiple, repeated comparisons between a potentially active substance vs. a placebo might also be recommended when investigating the individual ergogenic responses to other ergogenic substances/supplements.
